# Quercetin as an Emerging Anti-Melanoma Agent: A Four-Focus Area Therapeutic Development Strategy

**DOI:** 10.3389/fnut.2016.00048

**Published:** 2016-10-31

**Authors:** Zoey Harris, Micah G. Donovan, Gisele Morais Branco, Kirsten H. Limesand, Randy Burd

**Affiliations:** ^1^Department of Nutritional Sciences, University of Arizona, Tucson, AZ, USA

**Keywords:** melanoma, quercetin

## Abstract

Replacing current refractory treatments for melanoma with new prevention and therapeutic approaches is crucial in order to successfully treat this aggressive cancer form. Melanoma develops from neural crest cells, which express tyrosinase – a key enzyme in the pigmentation pathway. The tyrosinase enzyme is highly active in melanoma cells and metabolizes polyphenolic compounds; tyrosinase expression thus makes feasible a target for polyphenol-based therapies. For example, quercetin (3,3′,4′,5,7-pentahydroxyflavone) is a highly ubiquitous and well-classified dietary polyphenol found in various fruits, vegetables, and other plant products including onions, broccoli, kale, oranges, blueberries, apples, and tea. Quercetin has demonstrated antiproliferative and proapoptotic activity in various cancer cell types. Quercetin is readily metabolized by tyrosinase into various compounds that promote anticancer activity; additionally, given that tyrosinase expression increases during tumorigenesis, and its activity is associated with pigmentation changes in both early- and late-stage melanocytic lesions, it suggests that quercetin can be used to target melanoma. In this review, we explore the potential of quercetin as an anti-melanoma agent utilizing and extrapolating on evidence from previous *in vitro* studies in various human malignant cell lines and propose a “four-focus area strategy” to develop quercetin as a targeted anti-melanoma compound for use as either a preventative or therapeutic agent. The four areas of focus include utilizing quercetin to (i) modulate cellular bioreduction potential and associated signaling cascades, (ii) affect transcription of relevant genes, (iii) regulate epigenetic processes, and (iv) develop effective combination therapies and delivery modalities/protocols. In general, quercetin could be used to exploit tyrosinase activity to prevent, and/or treat, melanoma with minimal additional side effects.

## Introduction

Melanoma is an aggressive form of skin cancer that develops from neural crest-derived melanocytes. Due to its metastatic potential, melanoma is the leading cause of death of all skin cancer types, and its incidence and mortality has increased dramatically over the last 30 years. In the United States alone, 76,380 individuals are projected be diagnosed with melanoma in 2016 and 10,130 deaths are estimated to occur (1). The stage of melanoma determines the course of treatment. When diagnosed in its early states, melanoma can often be cured by surgery. However, when diagnosed at the metastatic stages, the prognosis is poor, with the 5-year survival rates of stages III and IV being 45 and 10%, respectively (2–4). As a result of new developments in cancer therapy, treatment at these stages now includes immunotherapy and targeted therapeutic drugs. Yet, despite these new developments, the response rate to such therapies (~10–20% for the immunotherapeutic agent, ipilimumab, and ~50% for the B-RAF inhibitor, dabrafenib) varies depending upon the molecular and genetic profile of the tumor (5–7). With the recent advancements in genomic sequencing, including RNA-seq, single-gene assays, and metabolomics, the unique molecular profile of tumors can now be identified rapidly and cost effectively. By investigating genomic variations, new molecular targets specific to a patient’s melanoma can be recognized and utilized for prevention, detection, and treatment.

Functional foods and dietary supplements as anticancer agents have gained interest because of their ability to increase the responsiveness of tumors to current treatments while keeping normal tissue toxicity low. Quercetin, a dietary polyphenol, is one bioactive compound that is of particular therapeutic interest because of its potential to both prevent and treat cancer. At low concentration (e.g., <100 μM), quercetin induces signaling cascades that lead to the induction of antitumor pathways. At relatively higher concentrations, quercetin can induce damaging cellular effects, including the production of pro-oxidant adducts and the alteration of glutathione (GSH) or bioreduction potential. Studies have also shown a selective sensitivity of melanoma tumor cells to the cytotoxic effects of quercetin (8), whereas normal tissues could be protected through antioxidant activity or the induction of protective cellular signaling pathways (9).

Melanocytes and melanoma cells, in particular, have a unique feature in that they specifically express the oxidative enzyme tyrosinase, which can oxidize quercetin into reactive adducts (10, 11). In this review, we propose that quercetin can be used as a basis for the development of a strategy for melanoma therapeutics. By exploiting the characteristics of quercetin and the expression of tyrosinase, therapies could be developed that specifically target melanoma and preserve or protect normal tissue. With modern genomic sequencing and drug delivery mechanisms, quercetin-based therapeutics could provide a great therapeutic advantage and positively impact prevention and treatment of melanoma. To use quercetin as an antitumor agent, several limitations must be overcome. This review proposes four focus areas to develop quercetin as anticancer compound. The four focus areas include utilizing quercetin to (i) modulate cellular bioreduction potential and associated signaling cascades, (ii) affect transcription of relevant genes, (iii) regulate epigenetic processes, and (iv) develop effective combination therapies and delivery modalities/protocols. By focusing on these areas, quercetin could be used to exploit tyrosinase activity, as well as prevent, and/or treat, melanoma with minimal additional side effects.

## Overview of Melanoma

Melanoma is characterized as a malignant tumor that often originates from pigment-producing melanocytes. Melanocytes are derived from neural crest cells, which are embryonic cells that have the ability to migrate to specific locations, where they can then differentiate into specific cells, including mature melanocytes (12). There are a large array of melanocyte populations, and their location spans multiple regions including the skin, meninges, mucosal surfaces, ear, and eye (13). The formation of melanoma then arises from a series of steps. The first step, known as the horizontal and radial growth phase, occurs when certain mutations in melanocytes lead to an increase in proliferative capacity (14). Progression to the vertical growth stage occurs when the altered melanocytes enter the dermis and/or hypodermis of the skin. Once the tumor cells invade the endothelium, they can travel to other locations within the body, thus leading to metastatic melanoma (15). Determinants of melanoma are influenced by both environmental (i.e., sunlight UV) and genetic factors. Such factors can influence the expression of the pigment-producing enzyme, tyrosinase, which has been shown to increase in tumors arising from melanocytes (16).

Several pathways are proposed to influence melanoma development, such as the RAS/RAF/MAPK, PI3K/AKT, and Notch pathways. Specifically, single base substitution mutations in two key genes involved in the MAPK signaling pathway, B-RAF and N-RAS, are commonly associated with development of melanoma (17–19). The most common oncogenic mutation in melanoma occurs in the serine/threonine kinase B-RAF gene at the 600 position, where a valine is replaced by either an arginine (V600K) or glutamic acid (V600E). Many studies have observed constitutive activation of ERK signaling in nude mice harboring the B-RAF^V600E^ mutation, leading to higher rates of proliferation and transformation (20–24). Although this mutation commonly occurs in melanoma, it should be noted that the mutation itself is not sufficient to cause cancer since it is also found in benign melanocytic lesions (17, 19, 25).

Likewise, the phosphoinositol-3-kinase–AKT (PI3K–AKT) pathway is also involved in melanomagenesis, and its activation often leads to increased cell survival, proliferation, and motility. Activation of this pathway in melanoma has been attributed to oncogenic mutations in the N-RAS gene as well as loss of expression or function of the tumor suppressor protein, PTEN (26). N-RAS mutations have been shown to activate the PI3K–AKT pathway *via* the direct binding to PI3K or through accumulation of activated RAS–GTP (27, 28). Although independent from N-RAS mutations, loss of PTEN is often found concurrently with the BRAF mutation mentioned above. Concurrent loss of PTEN with the BRAF mutation often leads to activation and cross talk between the MAPK and PI3K–AKT pathways (29). One study showed increased melanoma invasiveness in mice expressing melanocyte-specific BRAF^V600E^ with consecutive PTEN gene silencing, in comparison to mice expressing BRAF^V600E^ alone (30).

Involvement of the Notch pathway in melanoma development also plays an important role. Upregulation of the Notch receptors has been observed in malignant melanoma lesions, and activation of this pathway often leads to increased cell survival and growth (31). An *in vivo* study investigating the expression of Notch receptors in multiple uveal melanoma cell lines observed an increase in tumor growth, while suppression of the pathway utilizing short hairpin RNA segments that targeted the Notch2 receptor displayed a reduction in tumor growth (32).

In recent years, advances in the knowledge of the pathways described above and their role in metastatic melanoma have led to the development of new therapeutic agents. Until recently, the prognosis for advanced malignant melanoma was poor, and the only treatments approved by the Food and Drug Administration (FDA) were dacarbazine and IL-2. Even with these available treatment options, the 5-year survival rate and median overall survival were 6% and 7.5 months, respectively (4, 33). Recent advances in molecular profiling of tumors and immunotherapy have led to the development of new FDA-approved agents for metastatic melanoma, including the immune-checkpoint inhibitor, ipilimumab (34), and the BRAF inhibitor, vemurafenib (35). Ipilimumab’s mechanism of action allows for a prolonged antitumor T-cell response to malignant melanocyte antigens (34). One randomized, double-blind study evaluated the response of multiple doses of ipilimumab and found that a 10 mg/kg dose elicited a median overall survival rate of ~11 months (7). Other treatment options for metastatic melanoma include dabrafenib (36), another BRAF inhibitor used specifically in patients with the BRAF^V600E^ mutation, as well as trametinib (25), a MEK1/2 inhibitor used specifically in patients with the BRAF^V600E/K^ mutation. Table 1 shows current FDA-approved drugs for melanoma therapy including immune therapies, targeted therapies, and chemotherapeutics. For more information on the current treatments, we refer readers to the review by Maverakis et al. (37). Multiple phases II and III melanoma trials studying the effect of combination treatments are currently underway. However, due to the evolving resistance to such drugs and the adverse effects they carry, more effective combination treatments are still needed. Specifically, there is a need to prevent the induction of melanoma or develop combination therapies that target the unique molecular profile of melanoma tumors.

**Table 1:** **Current FDA-approved therapies for melanoma**.

Type of therapy	Drug name	Mechanism of action
Immunotherapy	Interferon alfa-2b	IFNAR/JAK/STAT activation
	Interleukin-2	Immune cell activation
	Ipilimumab	Anti-CTLA-4 monoclonal antibody
	Nivolumab	Anti-PD-1 monoclonal antibody
	Pembrolizumab	Anti-PD-1 monoclonal antibody
	Talimogene laherparepvec	Oncolytic viral therapy
Targeted therapies	Cobimetinib	MEK inhibitor
	Dabrafenib	BRAF^V600E^ inhibitor
	Trametinib	MEK-1/2 inhibitor
	Vemurafenib	BRAF^V600E^ inhibitor
Chemotherapy	Dacarbazine	DNA alkylating
	Temozolomide	DNA alkylating/methylating

*List of types of the therapy and specific drugs with corresponding mechanisms of action, currently in use for melanoma treatment*.

## Quercetin

The development of agents that produce limited side effects in prevention or therapy protocols is highly important. Dietary compounds as anticancer agents have gained attention because of the recent elucidation of their mechanisms of action. Flavonoids, for example, are a group of bioactive polyphenolic compounds that hold promise in the prevention and treatment of melanoma. More than 4,000 varieties of flavonoids are present in nature (38) and were first identified and isolated by Szent-Gyorgyin in 1936 (39). They are classified into seven main categories based upon variations in their heterocyclic C-ring, namely, flavones (e.g., apigenin, luteolin, and diosmetin), flavonols (e.g., quercetin, myricetin, and kaempferol), flavanones, isoflavones, catechins, anthocyanins, and chalcones (38, 40). Even though flavonoids are considered non-nutrients, they are important components of the human diet, presenting numerous beneficial health effects.

Quercetin derivatives account for 60% of the total flavonoids ingested daily and are the most abundant and important dietary flavonoids present in the human diet (41). The derivatives are commonly found in many vegetables and fruits, such as red onions, apples, berries (e.g., cranberries, strawberries, dark cherries, and blue berries), parsley, olive oil, cocoa, citrus fruits, tea, and red wine (42, 43). The estimated daily intake of quercetin varies according to food habits and can range between 5 and 40 mg a day, although these levels may rise up to 200–500 mg/day depending on the consumption of certain beverages, such as red wine and tea, in combination with a diet high in vegetables and fruits (44, 45). The potential toxicity of quercetin is quite low as human studies failed to demonstrate any adverse effects when quercetin was administered orally in single doses of 4 g or 500 mg thrice daily (46–48). Similarly, studies have reported no toxicity in humans with intake up to 1 g/day (42). This observation represents a critical factor that favors the utilization of quercetin in combination with standard cancer therapeutics.

**Figure 1:**
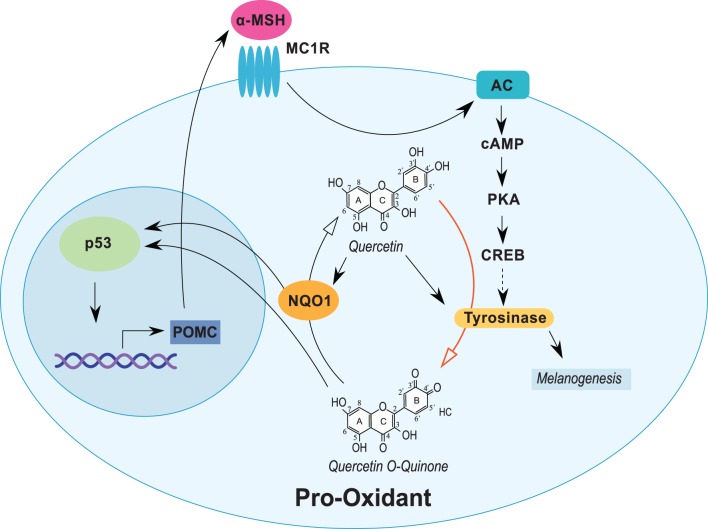
**Quercetin induces tyrosinase and stress response proteins in melanocytic cells**. Expression of tyrosinase in melanocytic cells is induced through the α-MSH pathway. Quercetin induces the expression of tyrosinase and several stress-responsive proteins, including NQO1 and p53. Tyrosinase oxidizes quercetin (red arrow) into an o-quinone and other reactive compounds that induce NQO1 and p53. NQO1 stabilizes p53 and can recycle activated quercetin back into the parent compound. Expression of p53 can also stimulate α-MSH activity completing a cyclical response to quercetin exposure. Open arrows indicate biochemical reactions. Closed arrows represent induction/stimulus.

Flavonoids are the most widely found compounds of plant phenolics. Their basic chemical structure is composed of diphenylpropanes (C6–C3–C6) with two aromatic rings linked through a pyran ring. Quercetin is classified as one of the best-described flavonoids. This compound possesses hydroxyl groups (–OH) attached to the 3, 5, 7, 3′, and 4′ positions (Figure 1). It may be present in plants and fruits in several different glycosidic forms in which one or more sugar groups are linked to phenolic groups by glycosidic linkage (49). For instance, quercetin forms the glycosides, quercitrin and rutin, together with rhamnose and rutinose sugars, respectively. Unlike quercetin glycosides, aglycone quercetin is not a normal dietary component.

The distribution, absorption, and metabolism of polyphenolic-containing foods, including quercetin, have been greatly studied in human and animal models in order to elucidate the biological activity and the ability of these compounds to enter cells. Quercetin is commonly found as a glycoside, which contains a sugar group bound to phenolic groups by glycosidic linkage (49). Present in a broad variety of vegetables, isoquercetin [quercetin 3-*O*-glucoside (Q3G)], represents a quercetin glycoside with a sugar at the 3-position (49). Aglycones (the forms lacking sugar moieties) occur less frequently.

Although not completely understood, numerous studies have shown that quercetin and other flavonoids are subject to hydrolysis and metabolic conversion during their absorption in the intestinal epithelial cell before reaching the bloodstream (49, 50). It is generally accepted that dietary quercetin glycosides are mainly hydrolyzed to the aglycone forms by intracellular β-glucosidases, which are subsequently converted into 3′-*O*-methylquercetin (isorhamnetin) and, to a smaller extent, into 4′-methoxyquercetin (tamaraxetin) (50, 51). It may also be sulfated or glucuronidated at one of the hydroxyl groups in the absorptive cells of the intestinal epithelium and the liver (52–54). Following this process, the resulting quercetin derivatives and any remaining unmetabolized quercetin are released into the circulation *via* the hepatic system.

Despite the difficulty in determining how quercetin is metabolized, several studies have confirmed that it reaches systemic circulation. Dietary studies in humans and animals have been performed and extensively reviewed (55); however, the number of studies is limited, and the quercetin administration protocol varies widely between studies, including differences in chemical composition of the compound administered, delivery medium, purity, and length of exposure. Pharmacokinetic studies in humans (56) and animals (57) demonstrate active metabolism and significant chemical modification. Additionally, consideration of the mixed polyphenols in the diet and their impact on absorption are important (58) as is the potential for synergy (59). Many polyphenols have anticancer properties with different mechanisms of action, bioavailability, and potency (60), and it is important to consider how multiple polyphenols interact.

In humans, Egert et al. administered 50, 100, or 150 mg/day quercetin orally for 2 weeks, and plasma levels were elevated by 178, 359, and 570%, respectively (54). Other studies conducted on animal and human models also confirmed the presence of quercetin metabolites and conjugates in blood samples (47, 61). Several studies demonstrate the formation of conjugates following metabolism. The most commonly observed metabolites in circulation involve conjugation with glucuronides and/or sulfates, and methylated forms of these metabolites. Glutathione conjugates have also been observed but are likely restricted to enzymatic activity in the liver (62). It is, however, important to consider what role cellular effects of these conjugates play, and how it may differ from pure quercetin is an important question.

Pertaining to melanoma prevention or therapy, the exact form and concentration of quercetin in skin are unknown. There is evidence that quercetin does have biological activity in skin. For example, quercetin mitigated radiation-induced skin fibrosis following oral administration in C3H/HeN mice (63). For detailed descriptions of the pharmacokinetics including bioavailability, metabolism, and biodistribution of quercetin and related polyphenolic compounds, we refer readers to the review articles by Cai et al. (64), and Kawabata et al. (65). We also refer readers to a review by Biasutto et al., which discusses potential insights and mechanisms to improve the bioavailability and bioefficacy of polyphenolic compounds (66). The limited number of clinical studies using quercetin that investigate oral bioavailability, the effect of supplementation protocols, and mechanisms of action, limits the ability to rationally design clinical protocols. Although quercetin holds promise, more clinical studies are required to fully realize the benefit of quercetin.

However, the rapid metabolism and low bioavailability is of concern especially with therapeutic applications. It is therefore proposed that long-term and low-dose dietary quercetin would be ideal for preventative protocols, but to achieve therapeutic applications, the compound must be used as an adjuvant and more technologically advanced delivery mechanisms must be utilized.

## Bioreduction Potential and Associated Signaling Cascades

Biological and pharmacological studies suggest that dietary polyphenols, such as quercetin, exhibit antioxidant, anti-inflammatory, antiproliferative, anti-obesogenic, anticancer, and other properties (11, 67, 68). In particular, the role of quercetin in anticancer activity has been established and extensively reviewed *in vitro* (69). Etiology and progression of many diseases are directly related to the oxidative stress generated by an imbalance between the formation and neutralization of pro-oxidants (70–74). Beneficial effects of quercetin on normal tissues have been attributed to several mechanisms, especially antioxidant effects. Within the flavonoid family, quercetin is the most potent scavenger of reactive oxidative species (ROS), and its anti-oxidative capacity is mainly due to the presence of phenolic hydroxyl groups on the B-ring and at the 3-position (49). At low concentrations, quercetin acts as an antioxidant by donating electrons to unstable ROS that have the potential to damage cellular DNA. Such damage can lead to mutations and influence the transformation of cancer cells that would be critically important in cancer prevention studies (11).

Quercetin’s anticarcinogenic effects have also been directly associated with its pro-oxidative properties in colon cancer, hepatoma, and melanoma cells (8, 75–77). Some studies have shown that the effects of quercetin are dependent on the concentration in tissue, mode of metabolism, and bioavailability (78). High concentrations of quercetin (e.g., 40–100 μM) likely promote pro-oxidant effects through oxidation of quercetin into o-quinone and the formation of reactive species, which culminate in apoptosis. Alternatively, low concentrations (<40 μM) have been shown to exert antioxidant properties (9, 79). Therefore, the biphasic nature of quercetin has the potential to be used as a dietary component for prevention of cancer at low doses and an adjuvant therapy to conventional cancer treatments at higher doses.

The biosynthesis of melanin and other pigments in melanocytes are catalyzed by tyrosinase, a copper-containing enzyme (80). In melanoma, the effect of quercetin can be amplified due to tyrosinase activity (Figure 1). Importantly, tyrosinase expression in melanocytic tumors increases during tumorigenesis (81). Enzymatic action catalyzed by tyrosinase utilizes quercetin as a substrate, which can form reactive o-quinone compounds (82). The activation of quercetin into quinone compounds has been observed in various cell lines, including melanoma cells (10, 11). Subsequently, after the formation of reactive species and metabolized quercetin, the compounds can bind to and deplete GSH, the main bioreductive antioxidant agent to prevent cellular damage (83). Additionally, the generation of ROS may directly trigger p53 induction and p53-mediated gene transcription and/or cell death through p53-independent apoptosis. The increase in ROS can also trigger endoplasmic reticulum stress (9) and the induction of mitochondrial proteins such as NOXA, p53, and PUMA with subsequent activation of procaspases 3 and 9 (84).

## Transcription of Relevant Genes

**Figure 2:**
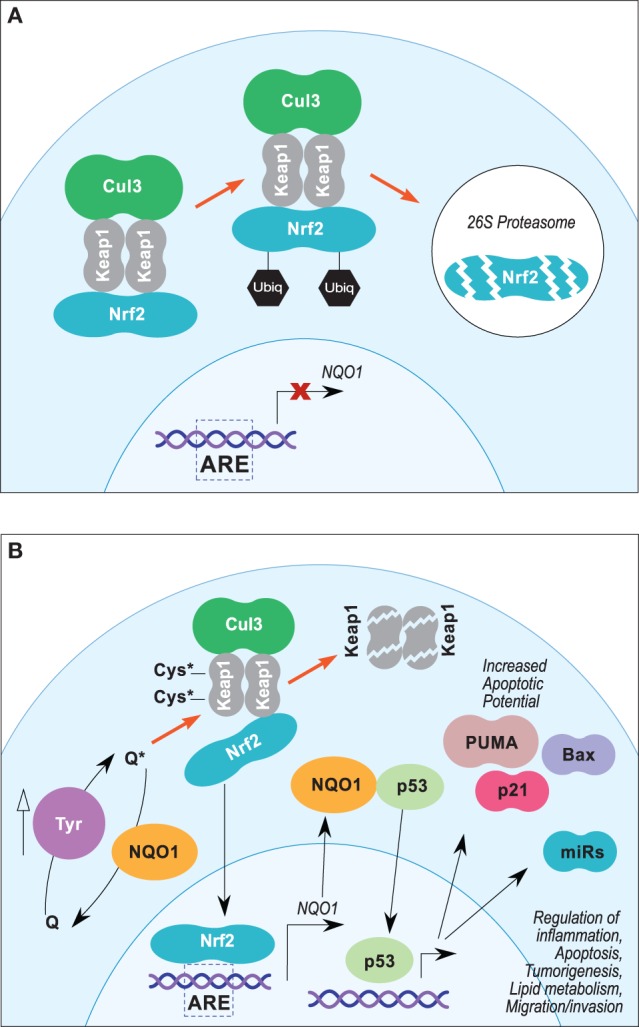
**(A,B)** Proposed Nrf2/ARE pathway leading to NQO1-mediated p53 stabilization in melanoma. **(A)** In steady-state conditions, Nrf2 transcriptional activity is suppressed by Keap1, which sequesters Nrf2 in the cytosol and facilitates Cul3-dependent ubiquitinylation and 26S proteasomal degradation. **(B)** Electrophilic stress at key cysteine residues of Keap1 leads to disassociation of Keap1/NRF2–DLG interface, which inhibits Cul3-dependent ubiquitinylation and promotes Keap1 degradation. Degradation of Keap1 protein allows accumulation of Nrf2 and translocation into the nucleus, which promotes expression of NQO1 and other genes under the control of AREs. Stabilization of p53 by NQO1 may potentiate expression of proapoptotic proteins and miRNAs.

The role quercetin plays in the induction of p53 is critical to anticancer therapies because of the potent transcriptional activity of p53 (Figure 2). In DB-1 melanoma cells overexpressing tyrosinase, quercetin administration led to significant increase of p53 protein and the number of cells in apoptosis compared to untreated cells (8). In particular, quercetin may potentiate p53-dependent apoptosis in melanocytic cells *via* stimulation of nuclear factor E2-related factor 2 (Nrf2) transcriptional activity, which has been observed in various cell types including human hepatoblastoma HepG2 cells (85, 86), human BJ foreskin fibroblasts and skin HaCaT keratinocytes (87), rat DI TNC1 astrocytes (88), and UVA-irradiated mouse B16F10 melanoma cells (89). Nrf2 is a basic leucine zipper (bZIP) transcription factor that induces the expression of several genes involved in cellular redox reactions, drug metabolism and transport, energy metabolism, and intracellular iron homeostasis in response to oxidative and electrophilic stress (90). A particular Nrf2 target gene of interest is *NQO1*, which encodes the cytoplasmic protein NAD(P)H dehydrogenase [quinone] 1 (NQO1) (90). NQO1 is a FAD-binding reductase that catalyzes two-electron reductions of quinone compounds, using NADH and NADPH as electron-donating cofactors (91). It has been demonstrated that NQO1 stabilizes p53 and prevents its ubiquitin-independent degradation (92–96) *via* a physical protein–protein interaction (92, 97). Given that the majority of melanomas (80–90%) express wild-type p53 proteins (98), upregulation of NQO1 by quercetin-mediated Nrf2 activity may provide potential means for targeted anti-melanoma therapy. Indeed, in DB-1 cells, upregulation of p53 was observed in parallel with increased NQO1 protein (8).

Nrf2 target gene activation is dependent on its nuclear translocalization and heterodimerization with small Maf proteins (90, 99). Nrf2–Maf heterodimers activate gene transcription by binding antioxidant response element (ARE) consensus sequences (5-^A/^G-TGA-^C/^G-NNNGC^A/^G-3) located in the regulatory regions of their target genes (99, 100). Nrf2 transcriptional activity is regulated in part by posttranslational ubiquitin-dependent proteasomal degradation and is induced in the presence of several electrophilic compounds, such as hydroquinones and quinones (91). Under normal conditions (Figure 2A), Nrf2 levels are constitutively repressed *via* Kelch-like ECH-associated protein 1 (Keap1)-dependent ubiquitylation by a Cullin-3 (Cul3) and RING-box protein 1 (Rbx1) E3 ubiquitin ligase complex (90). Under oxidative and electrophilic stress (Figure 2B), Nrf2 is stabilized, and its accumulation leads to increased ARE activation (90). Keap1 is an electrophile sensor protein that contains critical cysteine residues in the BTB (Cys151) and intervening region (IVR) (Cys273 and Cys288) domains (101). Modification of the nucleophilic cysteine residues of Keap1 by electrophilic Nrf2 inducers inhibits Cul3–Rbx1 ubiquitin ligase activity and stabilizes Nrf2 (90).

Single-particle electron microscopy data suggest a “hinge and latch model” for Nrf2 regulation by the Keap1/Cul3–Rbx1 complex (102). This model proposes that C-terminal Kelch-repeat domains of Keap1 homodimers bind a single Nrf2 protein at the DLG and ETGE motifs within the Neh2 domain (103). Binding at the Keap1–ETGE interface has ~2 orders of magnitude higher binding affinity than at the Keap1–DLG interface, which can be disrupted under oxidative and electrophilic stimulus (90, 103). An intact Keap1–DLG interface (“closed hinge”) puts Nrf2 in the optimal position for ubiquitylation by Cul3–Rbx1. Conversely, destabilization of the Keap1–DLG interface (“open hinge”) prevents the ubiquitylation and degradation of Nrf2 and increases ARE transcriptional activation (90). Although the exact mechanism remains to be fully elucidated, interruption of Keap1–DLG binding is likely the result of conformational changes brought on by modification of key cysteine residues of Keap1 by electrophilic Nrf2 inducers (90).

Quercetin has been demonstrated to induce Nrf2-mediated ARE activation *in vitro* in human hepatoblastoma HepG2 cells (85, 86), human BJ foreskin fibroblasts and skin HaCaT keratinocytes (87), rat DI TNC1 astrocytes (88), and UVA-irradiated mouse B16F10 melanoma cells (89). Tanigawa et al. demonstrated the ability of quercetin treatment to upregulate ARE transcription by regulating both Nrf2 and Keap1 in HepG2 cells (85). Quercetin treatment at a range of concentrations (5–40 μM) led to an increase in NQO1 protein levels, which was paralleled by increases in Nrf2 mRNA. This effect was attenuated by pretreatment with actinomycin D, which suggested that quercetin mediates Nrf2 expression, in part, at the transcriptional level. Quercetin also stabilized Nrf2 protein and decreased steady-state turnover by inhibiting Cul3–Rbx1-dependent ubiquitylation, which increased Nrf2 *t*_1/2_ reduction time by fourfold and suggests quercetin may prevent 26S proteasomal degradation of Nrf2. Finally, quercetin decreased levels of Keap1 protein but had no effect on Keap1 mRNA expression or ubiquitylation, suggesting it may downregulate Keap1 protein levels through a 26S proteasome-independent degradation mechanism. The upregulation of Nrf2 expression and stabilization of Nrf2 protein, coupled with decreased Keap1, leads to accumulation of Nrf2 in the nucleus and induction of Nrf2/ARE-activated target genes (85). Other studies in HepG2 cells suggest that the effect of quercetin on Nrf2 activation may be dose dependent (86). At lower concentrations (5–10 μM), quercetin significantly increased the nuclear translocation of Nrf2 and the nuclear content of phosphorylated Nrf2, whereas higher concentrations (50 μM) decreased both phospho-Nrf2 levels and the nuclear/cytosolic Nrf2 ratio (86).

Schadich et al. was able to demonstrate that quercetin administration in human BJ foreskin fibroblasts and aneuploid immortalized skin HaCaT keratinocytes led to significant upregulation of Nrf2 activity, as determined by a luciferase reporter assay (87). Nrf2 activity was increased in both cell lines treated with either 40 μg/mL of ginger extract or 30 μM of quercetin for 10 h. Western blot analysis showed increased levels of glutathione-*S*-transferase P1 (GSTP1) protein, a downstream target of Nrf2 activity, in BJ fibroblasts but not in HaCaT keratinocytes. It was suggested that the refractory response to upregulation of GSTP1 protein in HaCaT by activated Nrf2 is likely due to constitutively high expression in these types of cells, indicating a potentially distinct role of GSTP1 in HaCaT keratinocytes compared to normal cells (87). In immortalized rat DI TNC1 astrocytes, a range of quercetin concentrations (2.5–10 μM) attenuated lipopolysaccharide (LPS)-induced NF-κB activity and upregulated Nrf2 activity in both the presence and absence of LPS (88). Treatment with 10 μM of quercetin led to ~20-fold and 25-fold increase in Nrf2 activity in LPS-free and LPS-containing media, respectively, as determined by luciferase assay (88).

Loss of Nrf2 activity appears to play a role in melanogenesis (89). In primary human epidermal melanocytes and mouse B16F10 melanoma cells, siRNA-mediated silencing of Nrf2 enhances melanogenesis after UVA-radiation exposure (89). In B16F10 cells, UVA radiation alone significantly decreased Nrf2 nuclear translocation and ARE–luciferase activity 1 h post-irradiation. Pretreatment with quercetin (15 and 30 μM) 30 m before UVA exposure increased Nrf2 translocation and ARE–luciferase activity compared to control cells not treated with quercetin (89).

## Regulation Epigenetic Changes

Epigenetic alterations have recently been suggested to play a role in the initiation of carcinogenesis and induction of pro-cancer characteristics; thus, epigenetic modifying compounds have been proposed as anticancer agents (104–106). Quercetin has the potential to elicit significant epigenetic changes across multiple cell and tissue types (Table 2) (107–115). Specific epigenetic changes attributed to quercetin include changes in DNA methylation, histone acetylation, and micro-RNA (miR) expression. Although there is currently a lack of information regarding the epigenetic effects of quercetin specifically in melanocytes or melanoma, the epigenetic effects observed in other tissues likely play a role in the pathogenesis of malignant melanoma. Therefore, this area of research is critical to advancing prevention or therapeutic approaches.

**Table 2:** **Epigenetic activity elicited by quercetin**.

Epigenetic activity	Specific outcome	Experimental model	Concentration/dose	Exposure time	Analytical method	Reference
DNA methylation	DNMT inhibition	*In vitro* reaction	IC50 = 1.6 μM	30 m	^3^H-radioactivity assay	Lee et al. (107)
	DNMT inhibition	Human PC3 and DU145 prostate cancer cell lines	12 μM	24, 48, and 72 h	Colorimetric assay	Sharma et al. (108)
	CpG demethylation	Human PC3 and DU145 prostate cancer cells	12 μM	48 h	Bisulfite sequencing	Sharma et al. (108)
	CpG demethylation	Human RKO colon cancer cells	1 μM	120 h	MSP	Tan et al. (109)
	CpG demethylation	Human 9706 esophageal cancer cells	40 μM (nanoliposomal delivery)	48 h	MSP	Zheng et al. (110)
Histone modification	H3 acetylation	Human HL-60 leukemia cells	75 and 100 μM	3, 6, and 12 h	Western blot and ChIP assay	Lee et al. (111)
	HAT activation/HDAC inhibition	Human HL-60 leukemia cells	100 μM	6 h	Colorimetric assay	Lee et al. (111)
	HDAC inhibition	Human 9706 esophageal cancer cells	40 μM (nanoliposomal delivery)	48 h	Immunocytochemical assay	Zheng et al. (110)
	HDAC inhibition	Human HepG2 liver cancer cells	40 and 80 μM (nanoparticle delivery)	24 h	Colorimetric assay and Western blot	Bishayee et al. (112)
Micro-RNA expression	miR-155 downregulation	Murine RAW264.7 macrophages	10 μM quercetin and 10 μM isorhamnetin	6 h	Two-step RT-PCR	Boesch-Saadatmandi et al. (113)
	Hepatic miR-125b and miR-122 upregulation	C57B6/j mice	2 mg/g enriched diet	6 weeks	Two-step RT-PCR	Boesch-Saadatmandi (114)
	Hepatic upregulation of miR-467b, miR-374*, miR-30c-1, miR-450a-5p, miR-30b*, miR-197, miR-137, miR-466c-5p, miR-335-5p, miR-10b, miR-29a*, miR-196a, miR-7b, miR-190, miR-335-3p, miR196b, let-7c-2* and downregulation of miR-671-5p, miR-878-3p, miR-466f-3p, miR-486, miR-451, miR-144, miR-291b-5p, miR-324-5p, miR-296-5p, miR290-3p, let-7f*, miR-429, miR-298, let-7b*	Apo E^−/−^ mice	30 mg/day supplemental	2 weeks	Microarray analysis	Milenkovic et al. (115)

*Compilation of studies documenting quercetin’s effect on epigenetic activity*.

Inhibition and reversal of aberrant methylation on tumor suppressor genes may represent a significant target mechanism in cancer prevention and therapy (106). DNA methylation reactions are catalyzed by DNA methyltransferases (DNMTs), of which multiple forms are expressed in humans and other mammals (107). DNMT1 is the most abundant form expressed in humans and functions as a maintenance methyltransferase with high affinity for hemimethylated DNA. DNMT3A and DNMT3B are also expressed in humans and function as *de novo* methyltransferases with similar affinity to both hemimethylated and unmethylated CpG sites (116). Prokaryotic *SssI* DNMT is a bacterially derived *de novo* DNMT with functional similarities to DNMT3A and DNMT3B (107).

Quercetin inhibits both human DNMT1- and *SssI* DNMT-mediated DNA methylation in a dose-dependent manner (IC_50_ = 1.6 μM), as determined by an *in vitro* DNMT activity assay; however, this effect was dependent on the presence of catechol-*O*-methyltransferase (COMT) (107). Double-stranded dinucleotides (~2 mol CpG sites) were incubated with DNMTs, the methyl donor *S*-adenosyl-l-methionine (SAM, containing ~0.5 μCi of [methyl-^3^H]SAM), and a range of quercetin concentrations, and DNMT activity was determined by scintillation counting (107).

Quercetin has also demonstrated DNMT inhibitory effects in human PC3 and DU145 prostate cancer cells, RKO colon cancer cells, and Eca9706 esophageal cancer cells (108–110). The combination of quercetin (12 μM) and curcumin (14 μM) significantly decreased methylation at specific CpG sites within the AR promoter of both PC3 and DU145 prostate cancer cell lines, as determined by sodium bisulfite sequencing (108).

p16INK4α is an important tumor suppressor protein involved in cellular senescence by preventing CDK-dependent phosphorylation of Rb (117). Silencing of p16INK4α is associated with development of multiple tumor types, and the loss of the p16INK4α/Rb is suggested to contribute to melanomagenesis (118). Venza and colleagues investigated the epigenetic control of p16INK4α in clinical cutaneous (*n* = 60) and uveal (*n* = 6) melanoma tissue sections compared to normal skin samples (*n* = 48) (119). It was found that 63.33% (*n* = 38) and 50% (*n* = 3) of cutaneous and uveal melanomas, respectively, did not express p16INK4α mRNA, and 15% (*n* = 9) of cutaneous melanomas displayed expression levels below the cutoff point established in healthy samples. Of the 38 cutaneous samples with absent expression, 76.31% (*n* = 29) had aberrant homozygous hypermethylation within the p16INK4α promoter, and all 9 samples with low p16INK4α expression had heterozygous methylation (119). Another study using 59 clinical metastatic cutaneous melanoma samples found that p16INK4α was methylated in 25% of samples (*n* = 15) and that promoter methylation was significantly overrepresented in samples harboring *NRAS* mutations (120).

Administration of quercetin (1 μM) alone inhibited growth of human RKO colon cancer cells and restored p16INK4α gene expression in a dose-dependent manner, which was associated with a significant reversal of hypermethylation of the p16INK4α promoter (109). Similar effects have been observed in human Eca9706 esophageal cancer cells using nanoliposomal delivery of quercetin (40 μM), which suppressed cell growth, increased apoptosis, and increased p16INK4α expression (110). Increased p16INK4α expression was associated with decreased p16INK4α gene methylation, as determined by MSP, and decreased expression of DNMT1 (110).

Methylation of several other tumor suppressor genes has been observed to increase in melanoma including *RAR-b2, GATA4, WIF1, SOCS1, RASSF1A, TFP12, MINT17*, and *MINT 31* (121). It is likely that the methylation status of these factors and other tumor suppressor genes, including p16INK4α, are sensitive to treatment with demethylating agents such as quercetin, which may play a role in prevention and therapy of melanoma.

Histone acetylation, which refers to the addition of an acetyl group on specific amino acid residues on histone proteins, represents another epigenetic regulatory factor that may be targeted by quercetin in cancer prevention and therapy (122). Quercetin may also affect histone acetylation and subsequent gene expression by regulating histone acetyl transferase (HAT) and histone deacetylase (HDAC) activity (110–112). Activation of HATs by quercetin (100 μM) has been demonstrated in human HL-60 leukemia cells, which was associated with increased acetylation of histone 3 and FasL-related apoptosis (111). Treatment with quercetin also inhibited HDAC activity (111), which has also been observed in Eca9706 cells (40 μM) (110) and human HepG2 liver cancer cells (40 and 80 μM) (112).

Overall, evidence of the effect of quercetin on key cancer-driving epigenetic modifications in melanoma is lacking. However, given that epigenetic activity, such as aberrant methylation of tumor suppressor genes, is associated with melanomagenesis, this is a field that is largely open for further exploration to enhance preventative and therapeutic strategies.

## Combination Therapies and Delivery

**Figure 3:**
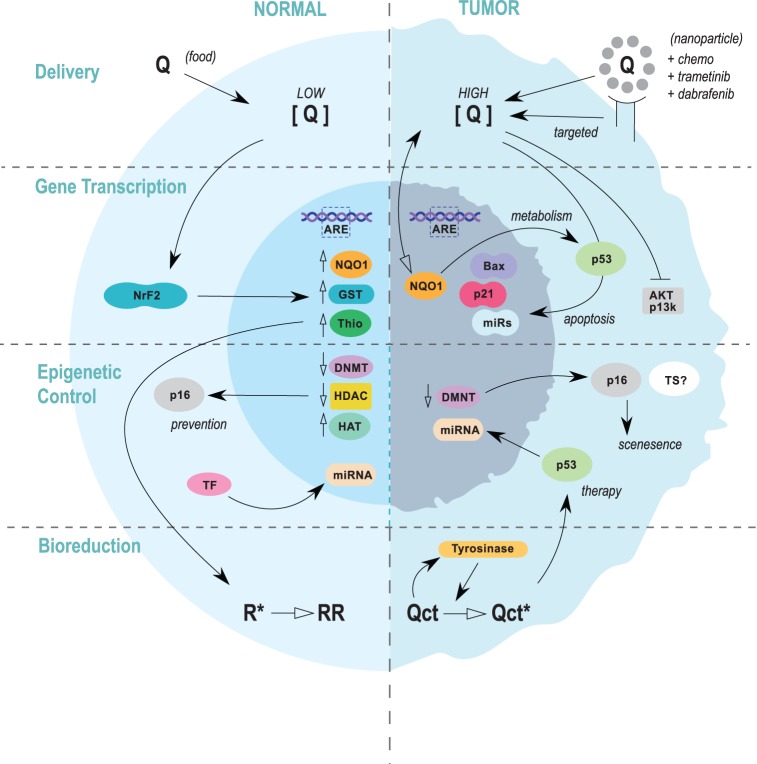
**Summary of the four focus areas to develop quercetin as a chemo-preventative or therapeutic agent**. In melanoma prevention scenarios (left), quercetin would be ingested through food sources and the concentration in normal melanocytes would be relatively low. Antioxidant activities and signaling pathways leading to the induction of cytoprotective proteins would dominate. Evidence suggests that quercetin may impart transcriptionally permissive epigenetic modifications within key tumor suppressor genes, including p16, which could confer resistance to oncogenesis. Induction of miRNAs could also aid in cancer prevention. In therapeutic protocols (right), quercetin would be delivered alone or in combination with anti-melanoma pharmaceuticals. Quercetin and additional compounds could be delivered through nanoparticles targeted to melanoma cells, or in an untargeted regimen. Pro-oxidant effects would be desired, and induction of wild-type p53 and other apoptotic factors would aid in therapy. Epigenetic mechanisms would likely be more prominent in prevention (blue line), but miRNAs have been shown to play a significant role in circumventing drug resistance.

Melanoma is an aggressive form of cancer that is refractory to current therapies. Additionally, as discussed above, bioavailability of quercetin is low, and as a food component, its anticancer potency is limited compared to pharmaceuticals. On the other hand, there are minimal side effects associated with dietary or systemic administration of quercetin, even at high levels. Thus, quercetin could be used in combination with other cytotoxic drugs, provided that bioavailability is addressed through chemical modification or use of a delivery system. Quercetin affects multiple signaling cascades and gene transcription, which also makes it a desirable adjuvant to biologicals. For example, there is new evidence suggesting the emergence of drug resistance to some of the newly approved FDA biological therapeutics in the treatment of advanced melanoma. Of particular interest are dabrafenib and trametinib. Dabrafenib is a selective inhibitor of the kinase, B-RAF^V600^, which causes upregulation of the MAPK pathway and is commonly mutated in over 50% of patients with metastatic melanoma (20, 123). This leads to further inhibition of B-RAF^V600^’s downstream targets, MEK1/2 and ERK, and thus decreased levels of cell survival and proliferation. Trametinib, on the other hand, functions to inhibit MEK1 and MEK2, also leading to reduced cell survival and proliferation. Resistance to these drugs has been attributed to activation of non-MAPK pathways (PI3K/Akt/mTOR), adaptive upregulation of Akt, overexpression of RTKs, and mutations in MEK (124–126). These mechanisms can significantly reduce the ability of both trametinib and dabrafenib to exert their antiproliferative effects and usually results in a reduced survival rate. Mechanistically, quercetin inhibits multiple pathways and could be an ideal candidate as an adjunct with current therapies, such as dabrefenib and trametinib (Figure 3). Quercetin (10–40 μM) displays dose-dependent attenuation of PI3K/Akt and MAPK signaling in UVB-irradiated B16F10 melanoma cells (127). This effect was observed in parallel with reduced cell viability, increased apoptosis, and enhanced nuclear translocation of NF-κB, a downstream effector in the PI3K/Akt pathway (127). A recent study in cervical cancer lines also demonstrated a dose-dependent decrease in levels of both phosphorylated PI3K and Akt, as well as of translocation of NF-kB (128). Similar effects were seen in another study where co-administration of quercetin and temozolomide decreased the levels of phosphorylated Akt more than temozolomide alone in glioblastoma cells (129). Quercetin was also shown to suppress the activity of PI3K through directly binding in an H-Ras-transformed MCF10A human breast epithelial cell line (130). These studies suggest that quercetin could be used in combination with current BRAF and MEK inhibitors to aid in the inhibition of pathways used for proliferation and survival in melanoma.

In addition to enhancing drugs that target signaling pathways, quercetin has shown efficacy in improving cytotoxic therapies such as temozolamide, which is commonly used to treat melanoma. For example, a recent study demonstrated that quercetin increased the effect of glioblastoma treatment compared to standard chemoradiotherapy alone through the inhibition of PI-3-kinase–Akt pathway. The study utilized cell lines, and the greatest reduction in cell viability and colony formation was observed when cells were treated with a combination therapy that included quercetin (129). Several studies have also addressed quercetin bioavailability in combination treatments. For example, a study analyzed liposomes loaded with quercetin and temozolomide to enhance the chemosensitization of drug-resistant cancer cells. The study demonstrated that DSPE-PEG2000 polymeric liposomes were an effective nanocarrier for enhancing drug delivery to tumors (131).

More novel approaches to compensate for the low bioavailability of quercetin have been made using liposomes, PLGA, PLA, chitosan, silica, and other compounds (69). Specifically designed nanosystems have proven to be effective at increasing bioavailability, such as the use of quercetin nanocrystals and nanoparticles. A recent *in vitro* study found that the efficiency of quercetin-loaded PLA nanoparticles was ~97%, and the nanoparticle encapsulation significantly improved the bioavailability of quercetin (132). Another study investigating the effects of nanocrystals found that its solubility was significantly higher compared to quercetin alone (133). Other important advances include the use of nanoparticles to improve solubilization of quercetin for increased oral uptake. For example, a quercetin-containing self-nanoemulsifying drug delivery system (Q-SNEDDS) was developed to increase quercetin oral bioavailability (134). Optimization of the delivery system significantly improved quercetin transport into cells grown as a monolayer. Oral ingestion resulted in rapid gut uptake following administration and resulted in peak plasma concentration, approximately twofold to threefold over control, 24 h after administration. Overall, the results suggested that Q-SNEDDS was a potent formulation that increased solubility and bioavailability through oral administration. Overall, the use of nanoparticles as a delivery platform for quercetin provides encouraging possibilities for therapeutic administration at high doses. Additional platforms can be used to co-deliver quercetin, such as co-administration of chemotherapies and drugs or biological therapeutics (135, 136), and can also be targeted to specific melanoma or tumor markers (Figure 3). These new emerging strategies of increasing quercetin bioavailability may further enhance quercetin’s effects in combination with current drugs that target tumor cells; however, more research and clinical testing is needed (69), especially in treatment protocol development to maximize tumor toxicity of the combinations and avoid any attenuation effects by the antioxidant properties of quercetin (137).

## Conclusion

Quercetin has great potential to be used an antitumor agent in melanoma, and various preventative and therapeutic options can be developed. This review has outlined four specific areas, that with further investigation, could facilitate the development of quercetin into an anticancer compound (Figure 3). The polyphenolic food compound has several desirable characteristics that can be exploited by targeting cells that express tyrosinase. The oxidation of quercetin can lead to a potentiation of its pro-oxidant effects such as an increase in p53 and Nrf2. There are several transcriptional events that result from quercetin treatment, which long term may aid in the prevention of melanoma or can be used to compliment current therapies. Activation of tyrosinase (oxidation) leads to enhancement of quercetin’s pro-oxidant effects and can also induce several signaling pathways, at least indirectly. Little is known about quercetin and its affect on epigenetic processes in melanoma, but observing other cancers as reference warrants investigation into this emerging area of prevention and therapy. Although data on quercetin’s influence on epigenetic changes in the skin and in melanoma are currently lacking, sufficient data exist to demonstrate quercetin’s capacity to elicit epigenetic changes in other tissues types.

Another future area that should be addressed is the induction of miRNA. Changes in miRNA expression have been suggested to play a role in human diseases such as cancer and cardiovascular disease among others (138). MiRNAs function to negatively regulate gene expression by repressing the translation of target mRNA sequences (139). Quercetin has demonstrated the capacity to affect miRNA expression in both cell culture and animal models. The effect of a quercetin-rich diet on miRNA expression has also been investigated in human lung cancer cases from the EAGLE case–control study (140).

Four focus areas were proposed to develop quercetin as a targeted anti-melanoma compound for use as either a preventative or therapeutic agent (Figure 3). Overall quercetin could be used to exploit tyrosinase activity to prevent, and/or treat, melanoma with minimal additional side effects. Dietary intake would be suitable in the development of preventative approaches, while nanoparticle systems will be required to achieve effective concentrations of quercetin for therapeutic approaches, likely as an adjuvant to melanoma-specific biologicals or possibly chemotherapeutics.

## Author Contributions

ZH is the author of the manuscript and participated in concept development. MD is the author of the manuscript, contributed equally with ZH, and participated in concept development. GB and KL participated in writing and concept development. RB participated in the writing, organization, and development of the overall manuscript concept.

## Conflict of Interest Statement

The authors declare that the research was conducted in the absence of any commercial or financial relationships that could be construed as a potential conflict of interest.

## Abbreviations

α-MSH, alpha-melanocyte-stimulating hormone; AC, adenylyl cyclase; ARE, antioxidant response element; Bax, Bcl-2-associated X protein; cAMP, cyclic adenosine monophosphate; CpG, cytosine–guanine dinucleotide; CREB, cAMP response element-binding protein; Cul3, Cullin-3; DNMT, DNA methyltransferase; ERK, extracellular signal-regulated kinase; GST, glutathione S-transferase; HAT, histone acetyltransferase; HDAC, histone deacetylase; Keap1, Kelch-like ECH-associated protein 1; MAPK, mitogen-activated protein kinase; MC1R, melanocortin 1 receptor; miR, micro-RNA; NQO1, NAD(P)H dehydrogenase [quinone] 1; Nrf2, nuclear factor (erythroid-derived 2)-like 2; PI3K, phosphatidylinositol-4,5-bisphosphate 3-kinase; PKA, protein kinase A; PLA, polylactic acid; PLGA, poly(lactic-co-glycolic acid); POMC, pro-opiomelanocortin; PTEN, phosphatase and tensin homolog; PUMA, p53 upregulated modulator of apoptosis; Q3G, quercetin 3-*O*-glucoside; Qct, quercetin; Rbx1, RING-box protein 1; ROS, reactive oxygen species; TF, transcription factors; Thio, thioreductase; Ubiq, ubiquitin.
